# Resistance to Innate Immunity Contributes to Colonization of the Insect Gut by *Yersinia pestis*

**DOI:** 10.1371/journal.pone.0133318

**Published:** 2015-07-15

**Authors:** Shaun C. Earl, Miles T. Rogers, Jennifer Keen, David M. Bland, Andrew S. Houppert, Caitlynn Miller, Ian Temple, Deborah M. Anderson, Melanie M. Marketon

**Affiliations:** 1 Department of Biology, Indiana University, Bloomington, IN, United States of America; 2 Department of Veterinary Pathobiology, University of Missouri, Columbia, Missouri, United States of America; Beijing Institute of Microbiology and Epidemiology, CHINA

## Abstract

*Yersinia pestis*, the causative agent of bubonic and pneumonic plague, is typically a zoonotic vector-borne disease of wild rodents. Bacterial biofilm formation in the proventriculus of the flea contributes to chronic infection of fleas and facilitates efficient disease transmission. However prior to biofilm formation, ingested bacteria must survive within the flea midgut, and yet little is known about vector-pathogen interactions that are required for flea gut colonization. Here we establish a *Drosophila melanogaster* model system to gain insight into *Y*. *pestis* colonization of the insect vector. We show that *Y*. *pestis* establishes a stable infection in the anterior midgut of fly larvae, and we used this model system to study the roles of genes involved in biofilm production and/or resistance to gut immunity stressors. We find that PhoP and GmhA both contribute to colonization and resistance to antimicrobial peptides in flies, and furthermore, the data suggest biofilm formation may afford protection against antimicrobial peptides. Production of reactive oxygen species in the fly gut, as in fleas, also serves to limit bacterial infection, and OxyR mediates *Y*. *pestis* survival in both insect models. Overall, our data establish the fruit fly as an informative model to elucidate the relationship between *Y*. *pestis* and its flea vector.

## Introduction

*Yersinia pestis* is the causative agent of bubonic plague, which is typically a rodent disease transmitted by fleas. Humans coming into close contact with infected animals or fleas may become accidental hosts, and the development of bubonic or septicemic plague ensues depending on the route of infection. In the most common form of infection, a fleabite deposits bacteria into the skin followed by colonization of the draining lymph node and subsequent formation of the characteristic swollen and painful bubo. Open wound exposure to *Y*. *pestis*, such as through contact with infected animals, may also deposit the bacteria directly into the bloodstream, causing the septicemic form of the disease. On rare occasions, the infection may progress to a secondary pneumonia, which can lead to person to person spread of the disease [1–3].

*Y*. *pestis* normally persists in sylvan rodent populations, where it is transmitted by fleas and may cause localized outbreaks [1,3]. Fleas become infected after taking a blood meal from an infected rodent [4]. An infected flea may survive for several weeks. During that time, the flea is able to transmit the disease to new hosts [5,6]. Transmission from flea to host is enhanced by formation of a *Y*. *pestis* biofilm in the flea midgut. Within 1–2 weeks of ingesting an infectious blood meal, the proventriculus of the Oriental rat flea, *Xenopsylla cheopis*, will become blocked by the biofilm. The blockage impedes entry to the gut, resulting in regurgitation of bacteria into the bite site. The unsuccessful feeding results in the flea attempting repeated blood meals, thereby increasing transmission of the disease [4,5]. The biofilm-dependent proventricular blockage may take up to two weeks to form, and the starving flea dies soon after becoming infectious [5,6]. However, there is evidence that transmission by other flea species such as *Oropsylla montana*, a major vector of plague in North America [7,8], may occur with incomplete or no blockage of the proventriculus [9–11].

A second mode of transmission, dubbed early phase transmission (EPT), appears to work through a biofilm independent mechanism [9,12]. Studies on a variety of rodents and fleas have detected transmission as early as one day after taking an infectious blood meal, and this was true even for biofilm deficient *Y*. *pestis* strains [9,11–17]. Both *O*. *montana* and *X*. *cheopis* fleas appear to have similar efficiencies of EPT [11]. Little is known about the bacterial factors that influence EPT, but several genes that contribute to long-term flea colonization and biofilm-dependent transmission are dispensable for EPT [11,18]. Based on the *O*. *montana* model, *Y*. *pestis* colonizes and can be transmitted immediately following a single infectious blood meal, allowing for rapid spread of disease during an outbreak [9]. After four days, transmission efficiency begins to wane [9], but maintenance blood meals on infected blood restores flea colonization and disease transmission [13]. It is plausible that simple mechanical transmission occurs, such as by contaminated mouthparts, but the contribution to disease spread by this route is unclear given evidence suggesting that *Y*. *pestis* does not survive long on these external surfaces [19]. Other mechanisms, such as midgut transmission or defecation have not been rigorously explored. Nevertheless, these mechanisms are likely to be influenced by interactions between the flea and bacteria that lead to the colonization of the midgut.

The immune response of insects is largely innate and particularly in the gut, reactive oxygen species (ROS), reactive nitrogen species (RNS), and antimicrobial peptides (AMP) are the primary effectors of host defense against the invading organisms [20–24]. Following a blood meal, fleas up-regulate expression of anti-bacterial defense mechanisms [25], and evasion of these is likely required for optimal vector colonization and disease transmission. Little is known about the genetic factors that enable *Y*. *pestis* to survive the initial onslaught of the insect immune system, however recent work implies that counteracting ROS and AMP production is important for flea colonization [25–29]. Unfortunately, the lack of complete genome sequences for important flea vectors or tools for genetic manipulation of fleas precludes direct testing of vector-pathogen interactions important to evasion of flea immunity.

In this study, we develop a model system using the fruit fly *D*. *melanogaster* to examine early colonization of the insect host by *Y*. *pestis*. *D*. *melanogaster* is one of the most highly studied genetic models, providing a distinct advantage over the experimental flea infection model. In addition, *D*. *melanogaster* has been used as a model system for numerous diseases [24,30–39]. Here we use a non-invasive, oral infection method [31] to establish a persistent gut infection of *Y*. *pestis* in *D*. *melanogaster* larvae similar to that of the flea. Furthermore, bacterial mutants with increased sensitivity to AMP and ROS yield decreased bacterial burdens in fly larvae. The attenuated phenotypes of these mutants in flies correlated with decreased bacterial survival in fleas, supporting a role for evasion of flea immunity in bacterial colonization. Thus, fruit flies may provide a useful model system in which to study events that influence vector-pathogen interactions.

## Materials and Methods

### Bacterial strains and media

*Yersinia pestis* KIM6+ (Pgm+ Hms+ Pla+) and KIM6 (Pgm^−^ (Δ*pgm*; *Hms* ^−^) Pla^+^) were received from R.D. Perry and are attenuated variants of *Y*. *pestis* biovar mediaevalis KIM, which lack the pCD1 plasmid [40]. KIM6+ *ymt*_H188N_ (from B.J. Hinnebusch) carries a point mutation rendering Ymt inactive [41]. KIM6+ Δ*phoP* and KIM6+ Δ*oxyR* were received from D. L. Erickson [42]. Δ*oxyR* was complemented with pJEToxyR [42]. Δ*phoP* was complemented with pLG*phoP* [27]. Construction of new strains and plasmids are described below. *Y*. *pestis* strains were propagated routinely on Heart Infusion Agar (HIA) supplemented with 0.2% galactose and 0.01% Congo Red to verify presence or absence of the *pgm* locus. Liquid cultures were grown in Heart Infusion Broth (HIB). For quantifying *Y*. *pestis* loads recovered from flies or fleas, insect homogenates were plated onto Yersinia Selective Agar (YSA) without supplements. *Y*. *pestis* cultures were grown at room temperature (24–28°C). *Escherichia coli* strains were propagated in Luria-Bertani agar or broth at 37°C. Antibiotics were added as appropriate to a final concentration of 20 μg/ml chloramphenicol, 100 μg/ml ampicillin, or 25 μg/ml kanamycin. Relevant plasmids and primers are listed in Table 1.

**Table 1:** Plasmids and primers used in this study.

Plasmid or primer	Relevant information	Citation
**Plasmids**		
pHSG576	Low-copy number bacterial expression vector	[43]
pBluescript	phagemid cloning vector	Stratagene
pFU34	Reporter gene fusion vector containing a promoterless *gfpmut3*.*1*	[44]
pAH118	pFU34 carrying the *npt* promoter to drive expression of GFP	This work
pAH121	pHSG576 carrying *Pnpt-gmhA*	This work
pKD13	Source of Km^R^ cassette	[45]
pWL204	Carries λ *red* recombination genes	[46]
pLB001	Source of Flp recombinase, created from pFLPs, Ap^R^	[47]
pTopo2.1YpFur	pCR2.1-Topo carrying the *Y*. *pestis fur* gene	This work
pUC18R6K-mini-Tn7T-Km	Delivery vector for mini-Tn7 carrying the kanamycin resistance cassette	[48]
**Primers**		
PKD13Kan.P1	5’-GTGTAGGCTGGAGCTGCTTC	[47]
PKD13Kan.P4	5’-ATTCCGGGGATCCGTCGACC	[47]
gmhA.P1	GAAGCAGCTCCAGCCTACACCATAAAGGAAGGCCTCTTTATATGTC	This work
gmhA.P2	tgtacgcgacatagcaggc	This work
gmhA.P3	acatttcacgggtgaaggg	This work
gmhA.P4	GGTCGACGGATCCCCGGAATTGATGTGCTGGGGTGATCAACC	This work
gmhA.SOE.3	gagacaggatgaggatcgtttcgcatgtaccacgatttaatccgtagtg	This work
gmhA.SOE.BamHI.4	ggatcctcaggcttttaccatctctttttc	This work
npt.pro.SOE.PstI.1	CTGCAGGCGCAAGGGCTGCTAAAG	[49]
npt.pro.gmhA.SOE.2	cactacggattaaatcgtggtacatgcgaaacgatcctcatcctgtctc	This work
gmhA.up	CCAGTGGCTCTTCAATCCC	This work
gmhA.down	GCGAGTATCGTTTAGTGCAAAG	This work
npt 5' int	TTGTCTGTTGTGCCCAGTCATAGCC	This work
npt 3' int	CGCTTCCTCGTGCTTTACGGT	This work
FurF	ACAGTAAACCTAGCAGTGCTTAAAA	[50]
FurR	TGAATCGATTGTAACAGGACTGAA	[50]
YpFurF	TCTGGAAGTGTTGCAAAATCCTG	[50]
YpFurR	AAGCCAATCTCTTCACCAATATCG	[50]
YpFurP	FAM-TGTCACCACGTCAGCGCGGAA GAT-BHQ1	[50]
qFurF	GCAACGGTTTACCGTGTTCT	This work
qFurR	CAGCTTGATGCCATGTTGTT	This work

### *gmhA* mutant construction

MEL44 (KIM6+ Δ*gmhA*) and MEL45 (KIM6 Δ*gmhA*) were created by λ Red-mediated recombination, as previously described [46,47]. The *npt* cassette flanked by FRT sites was amplified from pKD13 [45] using primers PKD13kan.P1 and PKD13kan.P4 [47]. Next, primer pairs gmhA.P1 + gmhA.P2 and gmhA.P3 + gmhA.P4 were used in two PCR reactions to amplify ~500bp of homologous regions on either side of *gmhA* from genomic DNA. The products of the three PCR reactions were combined to attach the *gmhA* homologous regions to the *npt* cassette. The resulting PCR product was electroporated into KIM6+ carrying pWL204 and expressing the λ red genes [46]. Proper insertion of the *npt* cassette was verified using the oligos gmhA.up, gmhA.down, npt 5’int, and npt 3’int. pWL204 was cured by growing colonies on 5% sucrose plates. The *npt* cassette was resolved by electroporating pLB001, which contains the Flp recombinase, into the strain [47]. Loss of *gmhA* was verified via PCR using the oligos gmhA.up and gmhA.down. pLB001 was in turn cured by growth on 5% sucrose. The final strain, MEL44, carries an unmarked deletion of *gmhA*. The same procedure was followed to create an unmarked deletion of *gmhA* in KIM6, called MEL45.

### Generation of kanamycin resistant *Y*. *pestis* strains for competition experiments

KIM6+ Km^R^, *oxyR* Km^R^, and *phoP* Km^R^ were created using pUC18R6K-mini-Tn7T-Km to generate kanamycin-resistant strains [48]. The mini-Tn7 delivery vector was electroporated into KIM6+, KIM6+ Δ*phoP* and KIM6+ Δ*oxyR*. Successful transformants were isolated by plating on HIA containing 50ug/ml kanamycin.

### Plasmid construction

GFP derivatives of *Y*. *pestis* strains were obtained through electroporation of plasmid pAH118, which was produced by cloning the constitutive *nptII* promoter fragment [49] upstream of the GFP open reading frame in pFU34 [44] using KpnI and BamHI. To complement the *gmhA* mutants, pAH121 was created as follows. The *gmhA* ORF was amplified with the *gmhA*.SOE.3 and gmhA.SOE.BamHI.4 primers, and the *npt* promoter from pAH118 was amplified with the npt.pro.SOE.PstI.1 and npt.pro.gmhA.SOE.2 primers. The products were combined and stitched together in another PCR reaction using the primers npt.pro.SOE.PstI.1 and gmhA.SOE.BamHI.4. The final PCR product containing *P*_*npt*_ *-gmhA* was cloned into the SmaI site of pBluescript and sequenced to verify the insert. *P*_*npt*_ *-gmhA* was removed by digesting with PstI and BamHI and then cloned into the corresponding sites in pHSG576 [43], creating pAH121. For qPCR validation, the *Y*. *pestis fur* gene was amplified from KIM6+ genomic DNA using primers FurF and FurR and then cloned into pCR2.1-TOPO vector (Invitrogen). The resulting plasmid called pTopo2.1YpFur was verified by sequencing the insert.

### Fly stocks and maintenance

The following *D*. *melanogaster* stocks were obtained from the Bloomington Drosophila Stock Center at Indiana University-Bloomington: *w* ^*1118*^ (Stock#5905), *imd* (Stock# 17474: y^1^ w^67c23^; P[EPgy2]imd^EY08573^), *duox* (Stock#16468: y1; P[SUPor-P]Duox^KG07745^/In(2LR)Gla, wg^Gla-1^ Bc1). Where indicated, wild type refers to *w* ^*1118*^. Stocks were maintained on standard cornmeal-agar medium at room temperature.

### Egg collection

Adult flies were placed in collection chambers capped with ethyl acetate (EA) plates (30 g. agar, 15 g. sucrose, 10 ml ethyl acetate per liter) or grape juice agar plates (25 g. agar, 250 ml grape juice, 6 ml propionic acid (or 17.2 ml Tegosept) per liter) dabbed with small amounts of yeast paste, prepared with sterilized yeast grain and water. After overnight incubation in the dark and at 25°C, fresh EA (or grape) plates and yeast paste were added and the flies were incubated at 25°C for 2–4 hours to ensure synchronized development of the eggs. After this incubation period, the plates were removed and the eggs on the plates were dechorionated by soaking in 10% bleach for 3 minutes followed by rinsing with sterile water for five minutes. The eggs were then placed in 6-well dishes containing 2 ml of solidified Bacto-Agar per well, and the wells were sprinkled with sterilized yeast pellets. The wells were sealed with a breathable membrane (Breathe-Easy, Diversified Biotech), and the plates were stored at room temperature overnight.

### Oral infection of fly larvae

Our procedure is based on the non-invasive infection method developed by Olcott et al [31]. On the day following egg collection and plating (Day -1) *Y*. *pestis* strains were inoculated into HIB with antibiotics and grown overnight at room temperature. On Day 0, the OD_600_ of bacterial cultures were measured, and doses were prepared by diluting the appropriate amount of bacteria into a killed yeast and sterile phosphate buffered saline (PBS) mixture to achieve a final concentration of 10^8^ CFU/ml of bacteria in 17% killed yeast. 0.1ml of the bacteria-yeast suspension was distributed over the surface of the appropriate well to initiate the infection. Uninfected control wells were fed an equivalent amount of bacteria-free yeast suspension. A portion of the bacterial suspensions were also diluted and plated in order to verify the initial inoculum. After the inoculation, wells were again sealed and larvae were incubated at room temperature. Except where indicated, larvae were harvested at daily intervals after infection (Days 1, 2, 3): five larvae from each well were retrieved and surface-sterilized by rinsing thoroughly with 70% ethanol and then water. Individual larvae were transferred to microfuge tubes containing 60 μl sterile PBS and homogenized with a pestle. The bacterial load per larva was assessed by either qPCR analysis (described below) or by plating homogenates onto medium in order to count colonies. For colony counts, homogenates were serially diluted in PBS, plated onto YSA (without supplements), and plates were incubated at room temperature for two days. Colonies were counted to determine the bacterial load per larva. For monitoring bacterial survival outside of the larvae, infections were performed as described above. Each day that larvae were retrieved for homogenization, agar samples were also taken. This was done by poking a pipette tip straight down through the agar and then rinsing the tip thoroughly in 150 μl sterile PBS. The suspensions were diluted further and then plated onto YSA as above.

### Statistical analysis for single-strain fly infections

For each experiment a single trial consisted of harvesting at least 5 larvae per bacterial strain per day. Because of biological and seasonal variation on larvae growth and infection, KIM6+ was included in every experiment, as the positive control and basis for comparison to bacterial mutants. Then, 2–3 separate trials were performed, and data were pooled to obtain a biological average (mean) and standard error of the mean (SEM), and these values were graphed. Statistical analysis was performed using one-way ANOVA with Dunnett's test, using KIM6+ infection of WT flies as the control.

### Quantitative PCR (qPCR) analysis of infected larvae

For some experiments involving *imd* flies, an unknown bacterial contaminant outcompeted *Y*. *pestis* for growth on YSA plates and prevented quantification of colony counts. To overcome that issue, bacterial loads in fly larvae were quantified using quantitative PCR (qPCR) analysis based on detection of the chromosomal *Y*. *pestis fur* gene as previously described [50]. In order to correlate qPCR data to CFU counts, a standard curve was constructed using the plasmid pTopo2.1YpFur, which contains the *Y*. *pestis fur* gene. Plasmid DNA concentration was determined using a NanoDrop (Thermo Scientific), then serially diluted to obtain 10^1^−10^8^ genome copies per μl and used for standard curve analysis using the Applied Biosystems StepOne Plus software. To measure bacterial loads in larvae, individual infected larvae were surfaced sterilized by rinsing with ethanol and then homogenized in 50 μl of lysis buffer from the Promega Wizard Genomic DNA Extraction kit. Kit instructions for Gram-negative DNA extraction were followed and DNA was suspended in 50 μl of sterile water. Each sample was analyzed in duplicate using either *Taq*Man or SYBR Green as described below.

For qPCR quantification using *Taq*Man, the analysis was performed essentially as described previously [50]. Detection was carried out in a final volume of 20 μl using 10 μl of 2X *Taq*Man master mix along with primers YpFurF and YpFurR at a final concentration of 800 nM, and YpFurP (a 5’ 6-fluorescein amidite (FAM) labeled probe with a 3’ Black Hole Quencher (BHQ1)) at a final concentration of 400nM, along with 5 μl of DNA template. The samples were analyzed with an Applied Biosystems StepOne Plus thermocycler using the following conditions: 95°C, 10 min followed by 45 cycles of 95°C, 30 sec and 60°C, 1 min. Bacterial abundance in the larval extracts was determined using the plasmid-based standard curve.

For qPCR quantification using SYBR Green, primer sets specific to the *Y*. *pestis fur* gene were designed using Primer 3 software [51] to yield 200 bp amplicons. DNA from infected larvae were analyzed with the following 20 μl reaction mixture: 5 μl template DNA, 10 μl of 2X Genecopeia SYBR Green Master Mix, 0.6 μl H_2_O, 2 μl of 2 μM qFurF, 2 μl of 2 μM qFurR, and 0.4 μl ROX dye. The Applied Biosystems OneStep Plus qPCR thermocycler was used with the following conditions: 95°C, 10 min followed by 40 cycles of 95°C for 10 sec; 60°C for 15 sec; and 72°C for 10 sec. Bacterial abundance in the larval extracts was determined using the plasmid-based standard curve.

### Competition experiments

To evaluate the fitness of the *oxyR* mutant, co-infections were performed. KIM6+ was used as the wild type reference strain for all co-infections. KIM6+ Km^R^ and *oxyR* Km^R^ carry a kanamycin resistance cassette in order to distinguish these clones from the reference strain in larval homogenates. Bacterial strains were grown to mid-exponential phase (OD_600_ = 0.5–0.7) and each was diluted to 10^8^ CFU/ml of bacteria in PBS with 17% killed yeast. Infectious doses were prepared using a mixture of the reference strain and decreasing amounts of the indicated tagged strain, while keeping the total bacterial concentration at 10^8^ CFU/ml. Flies were infected as described above using a total inoculum of 10^7^ CFU. On day 3 post-infection, bacteria were extracted from individual larvae, homogenized in PBS, and plated on YSA and YSA containing 50 μg/ml of kanamycin. Recovery of tagged strains was determined by the colony counts from YSA with kanamycin. Recovery of the reference strain was determined by subtracting the colony counts from YSA with kanamycin (i.e., tagged strain) from plain YSA (i.e., total bacteria). The fraction of tagged vs reference strain in the larval homogenates was calculated as the Log_10_ of the tagged strain CFU count divided by total CFU count. Each co-infection experiment was conducted twice, and the data from both trials were pooled to give n = 15 per co-infection mixture. Six mixtures were tested (ranging from 1:1 to 1:100,000) for each set of co-infections.

### Visualization of bacterial niche within larvae

To visualize pH along the gut, 0.05% bromophenol blue in LB with 5% sucrose was used in place of PBS in the yeast or yeast+bacteria suspensions. The mixture was fed to larvae as described above. For monitoring localization of bacteria, *Y*. *pestis* strains carrying pAH118 (GFP) or *E*. *coli* carrying pRSETb-mCherry [52] were used to infect larvae. In the cases of co-infection, equal amounts of bacterial strains were used to prepare the inoculum for a total of ~10^9^ CFU. Larvae were rinsed and placed on slides for immediate imaging with an Olympus SZX16 Research Stereoscope equipped with an Olympus DP72 camera and labSens software.

### Ethics Statement

All procedures were performed according to the NIH Guide for the Care and Use of Laboratory Animals and were approved by the University of Missouri Animal Care and Use Committee (Protocol #6501) in accordance with the Animal Welfare Act. All efforts to minimize pain and suffering as well as to reduce animal numbers have been made. Hypothermia-anesthetized neonatal mice were used to provide fresh blood meal to the fleas. Neonates were subsequently euthanized by decapitation.

### Flea infections

*Oropsylla montana* and *Xenopsylla cheopis* fleas were maintained in a refrigerated incubator at 22°C with ~80% relative humidity [5]. Fleas were reared in one gallon glass jars containing larval medium (3 parts saw dust to 1 part of an equal blood meal, dried milk, mouse pellet mixture). For infection, fleas were starved for 6 days and subsequently allowed to feed on 3-4ml of heparinized murine blood containing 10^9^ CFU of *Y*. *pestis* [53]. Following infection, fleas were anesthetized using a chill table set to 0°C and monitored under a dissection scope for signs of fresh blood in the esophagus or proventriculus. Fleas that did not acquire a *Y*. *pestis*-infected blood meal were removed from the study As previously shown, fleas of either species infected by this method resulted in 50–95% of fleas that took up an infectious blood meal, and of those that ingested a blood meal, typically 90% were infected [9,11]. To verify equivalent ingestion of inoculating dose for all *Y*. *pestis* strains, 5 fleas were collected per strain and individually homogenized for enumeration of bacterial load (S2 Fig and data not shown). Mechanical disruption of fleas for bacterial enumeration was performed using a bead beater and homogenates were plated in duplicate on HIA. Statistical analysis of clearance rates (% of fleas not infected) for each mutant compared to KIM6+ was performed using Chi-square test (significance was defined as α<0.05) with Bonferonni post-hoc test for multiple comparisons (significance was defined as P<0.025). To analyze bacterial burdens in fleas that remained colonized, a non-parametric rank analysis was performed using a Kruskal-Wallis test (significance was defined as α<0.05) with Dunn's post-hoc test for multiple comparisons.

## Results

### *Y*. *pestis* colonizes *D*. *melanogaster* larvae

To determine whether *Y*. *pestis* could colonize and persist within the larval fruit fly, we employed the oral infection strategy of Olcott et al [31]. *D*. *melanogaster* larvae were fed a suspension of heat-killed yeast seeded with *Y*. *pestis* strain KIM6+. We monitored bacterial growth within insects as well as on the agar medium. To evaluate the robustness of this infection strategy, we tested a variety of inoculum doses used to seed the yeast suspension, ranging from 10^3^ to 10^9^ CFU of *Y*. *pestis*.

**Fig 1:**
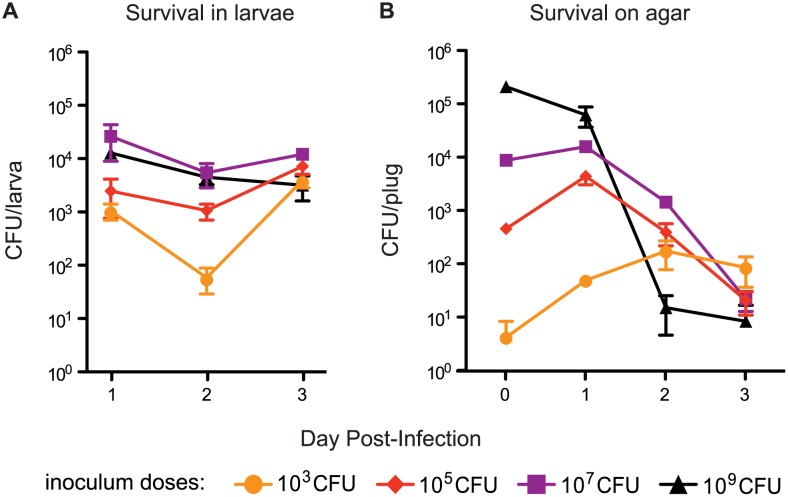
*Y*. *pestis* is able to colonize the midgut of *D*. *melanogaster* larvae. One day after hatching, larvae were infected with the indicated inoculum of *Y*. *pestis* KIM6+. Bacterial loads were evaluated in two ways at the indicated times post infection: **(A)** Means and standard error from five individual larvae at each time point that were surface-sterilized and homogenized; **(B)** Means and standard error from three agar samples that were taken at each time point and suspended in 150 μl sterile PBS. The bacterial suspensions from both methods were serially diluted, plated on YSA, and incubated at room temperature for two days before counting to determine bacterial survival within larvae or on the agar plates. Data is representative of at least three independent trials.

Regardless of inoculum, the initial bacterial burden on day 1 was between 10^3^ and 10^4^ CFU/larva (Fig 1A). Overall, colonization levels were stable over the three-day time course, which represented late first/early second instar through third instar stages of larval development. On days 3 to 4, larvae typically began to pupate, which marks the transition from the larval stage to the adult stage, and *Y*. *pestis* infection did not cause any apparent delays in reaching this developmental landmark. All infected larvae appeared normal and healthy. Bacterial colonization of developmental stages beyond the onset of pupation was not investigated due to institutional biosafety restrictions regarding adult flies. In contrast to survival inside larvae, the bacterial survival on the agar medium tended to decrease over the course of the infection (Fig 1B). There was some growth observed on the medium inoculated with the lowest dose, but overall the trends suggest that nutrient availability on the agar is not sufficient to support robust growth outside of the larvae. These data indicate that *Y*. *pestis* persistently infected fruit fly larvae.

### *Y*. *pestis* localizes to the anterior midgut of larvae

**Fig 2:**
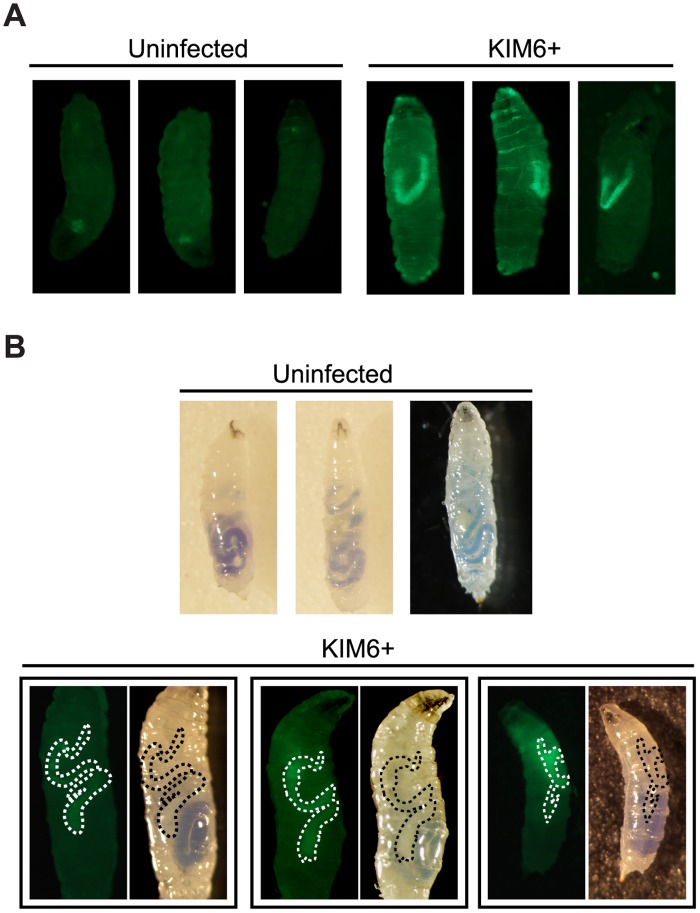
Localization of *Y*. *pestis* within an infected larva. (A) The KIM6+ GFP reporter strain (10^9^ CFU) was used to infect larvae, and bacterial localization was visualized using fluorescence microscopy. Representative images from three different larvae are shown (n>30 from at least three independent trials). (B) Larvae were infected as in panel A, except that 0.05% bromophenol blue and 5% sucrose were included in the larval food in order to visualize regions of the gut. Acidic = yellow; neutral = clear; alkaline = blue. Larvae were imaged using standard bright field and fluorescence microscopy. Representative images are shown for uninfected (n = 28) and infected larvae (n = 25) from two independent trials. For infected larvae, dashed lines outline the bacterial niche, which was determined based on the GFP signal.

While the above data suggest that *Y*. *pestis* colonizes fly larvae, it is not clear if the bacteria are located within the gut and if this is a stable infection. To determine localization of the bacteria within larvae, we repeated the infection using *Y*. *pestis* KIM6+ expressing GFP. Bacterial colonization was monitored by imaging infected larvae followed by bacterial enumeration of larval homogenates. By day 1 post infection, the bacteria appeared to localize to the same region of most larvae, which was indicative of a niche within the gut (Fig 2A). This trend persisted on day 2 and 3 post-infection, although much of the bacterial signal was obscured by larval auto-fluorescence on day 3 (data not shown). The stable fluorescent signal is consistent with the stable bacterial burden over the three-day time course (Fig 1A and data not shown).

Though the pattern of fluorescence observed in Fig 2A suggested that the bacteria were localized to a section of the gut, we wanted to confirm the anatomical location of the *Y*. *pestis* niche. To do this, bromophenol blue was added to the bacteria-yeast suspension that was fed to larvae. The dye ranges from yellow in acidic conditions, to clear in neutral conditions, and blue in alkaline conditions [54]. We monitored infected larvae using bright field microscopy to assess anatomy and pH of the digestive tract and fluorescence microscopy to track the presence of GFP-expressing bacteria. Fig 2B shows that both uninfected and infected larvae ingested the food and showed similar overall gut architecture. When the bright field and fluorescent images were compared, *Y*. *pestis* localization in the fly larva was clearly enriched in sections of the gut that were relatively neutral (Fig 2B, regions are outlined in paired images), which is similar to the estimated pH of 6–7 for the flea midgut [2,55]. Overall, these data confirm that *Y*. *pestis* colonizes the fruit fly larval gut. Furthermore, the data indicate that, as in fleas, *Y*. *pestis* infection is not invasive and is primarily confined to the anterior midgut.

### *Y*. *pestis* localization to the anterior midgut is specific

**Fig 3:**
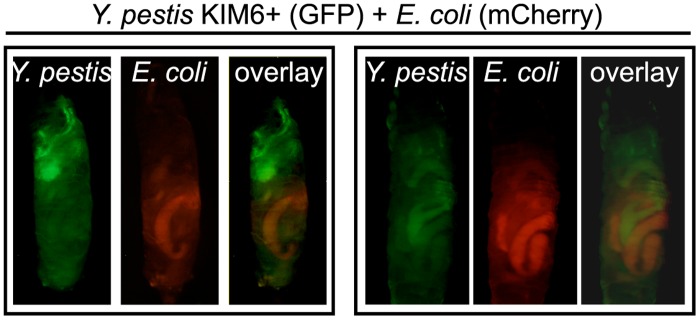
Localization of *Y*. *pestis* to the midgut is specific. Larvae were coinfected with ~5x10^8^ CFU each of KIM6+ strain expressing GFP (green) and *E*. *coli* containing mCherry (red). Larvae were imaged using fluorescence microscopy to detect both GFP and mCherry and images from both fluorescent channels were overlaid. Representative images from two trials are shown (n = 15 larvae).

The above data indicate that *Y*. *pestis* bacteria fill a niche in the anterior midgut; however we questioned whether this was a passive, transient association with the host or whether the *Y*. *pestis* niche is a stable, specific occupation. To address this question, *D*. *melanogaster* larvae were co-infected with KIM6+ expressing green fluorescent protein (GFP) and *E*. *coli* DH5α expressing mCherry, a red fluorescent protein. As before, *Y*. *pestis* was consistently enriched in the anterior midgut (Fig 3). In contrast, *E*. *coli* primarily localized to a posterior portion of the larval digestive tract. When the images are overlaid, it is apparent that *Y*. *pestis* and *E*. *coli* prefer to occupy distinct regions of the gut during infection. These data are consistent with the idea that *Y*. *pestis* establishes a stable niche, specifically within the anterior midgut.

### *Y*. *pestis* mutants lacking host immune defense factors show decreased levels of larval infection

**Fig 4:**
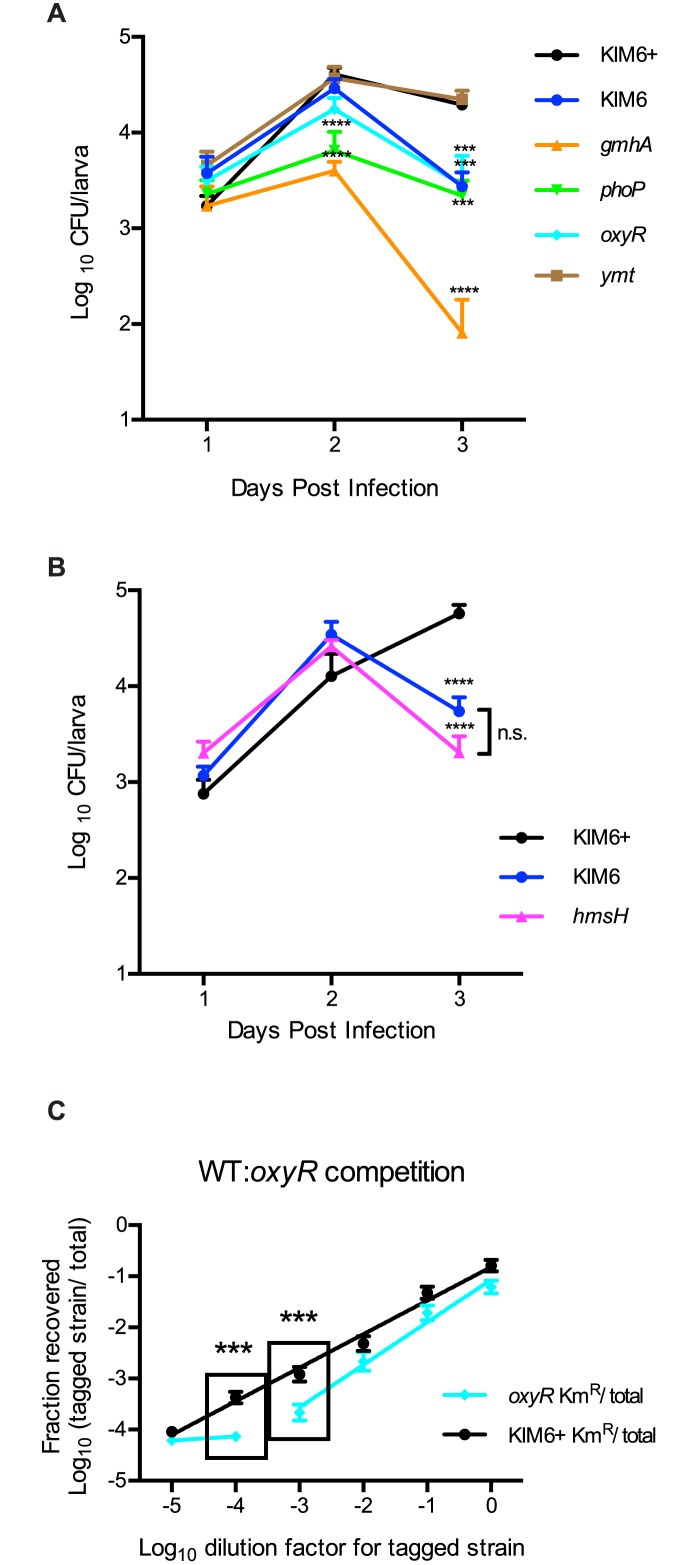
Decreased survival in flies of *Y*. *pestis* mutants. (A) *Drosophila* larvae were infected with the indicated strain of *Y*. *pestis* one day after hatching. Bacterial survival rate was determined by homogenizing larvae and plating on YSA on the indicated day post infection. Cultures were incubated at room temperature for two days before colonies were counted. For each trial, infections were performed in triplicate, and each strain was subjected to a minimum of three independent trials. Data from trials were pooled and averaged. Means (n≥15 per strain per day) and standard error are shown. ANOVA with Dunnett post-test was done to compare mutants to the KIM6+ control for each day. *** P<0.001, **** P<0.0001. (B) Larvae were infected and bacterial survival was evaluated as in Panel A. Infections were performed in triplicate (n = 15 per strain per day). Means and standard error are shown. ANOVA with Bonferroni multiple comparisons post-test was performed to make comparisons between strains for each day. **** P<0.0001 compared to KIM6+, n.s. = no significance between mutants. (C) Wild type *Drosophila* larvae were infected with mixtures of WT *Y*. *pestis* (KIM6+ reference) and either KIM6+ Km^R^ or *oxyR* Km^R^. After three days, the abundance of each strain per larva was enumerated in order to calculate the fraction of the kanamycin-resistant tagged strain out of the total bacterial load in the output. Those means and standard error were plotted against the dilution factor of the tagged strain relative to the reference strain in the input, and a linear regression analysis was performed to fit the data points. Each experiment was repeated twice (n = 15 for each data point). Black line = KIM6+ vs KIM6+ Km^R^ infections. Blue line = KIM6+ vs *oxyR* Km^R^ infections. ANOVA with Bonferroni post-test was performed to compare the means of the *oxyR* mutant competition (blue data points) to that of the WT competition (black data points) at each dilution step. Boxes indicate comparisons that were significantly different. *** P<0.001.

In the fly gut, the immune response is driven primarily by the Imd pathway, which induces AMPs such as diptericin [32,56]. In addition, the NADPH oxidase, Duox, is responsible for production of ROS, which serves as another major component of innate immunity in the gut [32,57]. Bacterial mutants lacking the genes necessary for an effective defense against these host responses should be defective for colonization. OxyR is a transcriptional regulator controlling expression of peroxidases and catalases in *Y*. *pestis*, and consequently the mutant is unable to defend against ROS [42,58]. PhoP is another transcriptional regulator whose regulon includes lipo-oligosaccharide (LOS) modification genes that are necessary for AMP resistance [27,42,59]. To test our hypothesis, *D*. *melanogaster* larvae were infected with the previously characterized *phoP* and *oxyR* mutants [42]. We observed that both mutants were significantly attenuated for fly colonization (Fig 4A). Larvae infected with the *phoP* mutant initially displayed bacterial loads similar to KIM6+ infected larvae, but by days 2 and 3 an approximate 10-fold lower bacterial burden was observed. Likewise, though the *oxyR* mutant colonized to a similar extent as wild type on day 1, by day 3 the mutant bacterial burden had dropped about 10-fold compared to wild type. Notably, complementation of both the *phoP* and *oxyR* mutants with plasmid-borne copies of the respective genes restored wild type levels of larval colonization (S3 Fig). As a control, we infected larvae with a previously characterized *ymt* mutant, which is severely deficient in colonizing the flea midgut [60]. Ymt is a phospholipase that appears to be important for detoxification of a component generated during digestion of the blood meal in fleas [60]. Because a blood meal is not involved in the fly infection model, and because loss of *ymt* has no apparent effect on sensitivity to AMP, ROS, pH or other stresses expected of the insect gut [61], we did not expect this mutant to be defective in our colonization model. Indeed, the *ymt* mutant behaved identically to wild type (Fig 4A).

### Contribution of *Y*. *pestis* biofilms to colonization of the fruit fly gut

In the flea model, biofilms have been shown to contribute significantly to persistent colonization of the flea midgut. However, mutants lacking exopolysaccharide (EPS) biosynthesis required for biofilms can still colonize fleas and are still effective in disease transmission [9,11]. To determine whether biofilms contribute to *Y*. *pestis* colonization within the fly midgut, we evaluated several known biofilm-deficient mutants. *Y*. *pestis* strain KIM6 lacks the *pgm* locus, which encompasses the *hmsHFRS* operon required for EPS synthesis, and is unable to form biofilms. *Y*. *pestis gmhA* was previously identified from a screen utilizing *Caenorhabditis elegans* to investigate biofilm formation, in which the mutant was unable to form a biofilm on the worm exterior [62]. The biofilm defect of the *gmhA* mutant was also observed in fleas, since the mutant was capable of colonizing fleas without establishing the biofilm blockage [62]. When we introduced these mutants to fly larvae (Fig 4A), we found that biofilm production does contribute modestly to colonization as demonstrated by the ~10-fold reduction in bacterial burden for the KIM6 strain compared to the KIM6+ parent. Furthermore, an *hmsH* mutant displayed a phenotype identical to the KIM6 mutant (Fig 4B), indicating that the *hms* operon within the *pgm* locus is the contributing factor for wild type colonization levels. Interestingly, the *gmhA* mutant was severely attenuated in fly larvae. The mutant yielded a 4-log decrease in the average bacterial load per larva compared to wild type (Fig 4A). This was unexpected, since the mutant showed only a defect in biofilm formation, not colonization, in fleas [62]. However, the differences in phenotypes could stem from the time period of the two infection models. In the previous study [62], bacterial burdens within fleas were assessed on days 7 and 28, whereas our study assessed fly colonization over the first three days of infection. Notably, colonization of the *gmhA* mutant could be restored to near wild-type levels with a plasmid-borne copy of the gene (S3 Fig).

### ROS-sensitive mutant is less competitive in vivo

We next assessed whether a modest colonization defect, such as that displayed by the *oxyR* mutant, would translate into reduced fitness when the mutant is forced to compete against wild type *Y*. *pestis*. To perform competitions, wild type *Y*. *pestis* was used as the reference strain and was mixed with varying amounts of a strain tagged with kanamycin resistance. The mixtures of strains (the "input") were used to infect fly larvae, and after three days of infection, bacterial loads in the larvae (the "output") were assessed. As expected for equally fit strains, when tagged wild type *Y*. *pestis* was competed with the untagged wild type strain, there is a corresponding linear reduction in recovery of the tagged wild type as it is diluted in the input (Fig 4C, black line). Kanamycin resistant colonies were consistently recovered through the dilution series until a dilution factor of 10^−4^ (a 1:10,000 ratio of tagged:untagged), beyond which the tagged strain was cleared from most flies. The constant, linear decrease in recovery of the tagged wild type strain is in contrast to the trend observed when the *oxyR* mutant was competed against wild type *Y*. *pestis* (Fig 4C, blue line). In that case, the initial decrease was linear, but the slope was steeper compared to wild type line (P = 0.0123), indicating that the mutant was outcompeted and cleared at a higher rate than wild type. This difference in mutant recovery became significant at a dilution factor of 10^−3^ and by 10^−4^ the mutant had already reached its limit of detection. From these results, we conclude that while the *oxyR* mutant grows as well as wild type in vitro (data not shown), it appears to be less fit in insects (Fig 4A), which translates into a competitive disadvantage in mixed infections (Fig 4C).

### The colonization defect of the *gmhA* mutant is multifactorial

GmhA is a phosphoheptose isomerase, which catalyzes the first step in the heptose biosynthesis pathway, and is therefore required for attachment of distal sugars to the LOS inner core [63]. Though the *gmhA* gene has a demonstrated role in biofilm formation in *C*. *elegans* and fleas [62], lack of biofilm formation alone cannot account for the severe colonization defect we observed in fly larvae. Based on the data in Fig 4, we infer that AMP and ROS resistance also contribute to colonization. Importantly, mutations leading to altered LPS and LOS often lead to AMP sensitivity [28,62,64–66]. To further investigate the basis of the *gmhA* mutant attenuation, we assessed biofilm formation and AMP resistance separately using in vitro assays. To evaluate biofilms, we incubated bacterial strains in borosilicate test tubes overnight at room temperature followed by crystal violet staining to quantify adherent biomass (S4 Fig). In this assay, the *gmhA* mutant produced 2.5-fold less biofilm than wild type, while the biofilm null mutant KIM6 produced 3.4-fold less biofilm than wild type. The residual amount of biofilm was eliminated when the *gmhA* mutation was introduced into KIM6. Complementation of *gmhA* in KIM6+ was able to recover wild type biofilms, while complementation of *gmhA* in KIM6 had no effect. These results confirm that *gmhA* contributes to biofilm formation in vitro, and we infer that *gmhA* is required for additional processes, such as AMP resistance, in vivo.

A suite of cationic AMPs, such as diptericin, cecropins and attacins are produced in the digestive tract of adult and larval flies [56,67]. To assess whether the *gmhA* mutant colonization defect could be attributed to increased AMP sensitivity, we next examined survival of the mutant when grown in the presence of the cationic AMP polymyxin B (S5 Fig). The *phoP* mutant, which cannot defend against cationic antimicrobials [27,42,59], was included as a positive control for AMP sensitivity. As expected, the *phoP* mutant was extremely sensitive to polymyxin B and could not tolerate levels beyond 1 μg/ml. Interestingly, the *gmhA* mutant was also sensitive to the AMP and could not grow at concentrations higher than 5 μg/ml. From these data, we conclude that the colonization defect of the *gmhA* mutant likely stems from the combination of reduced biofilm formation and increased AMP sensitivity.

### Rescue of AMP-sensitive bacterial mutants in vivo

**Fig 5:**
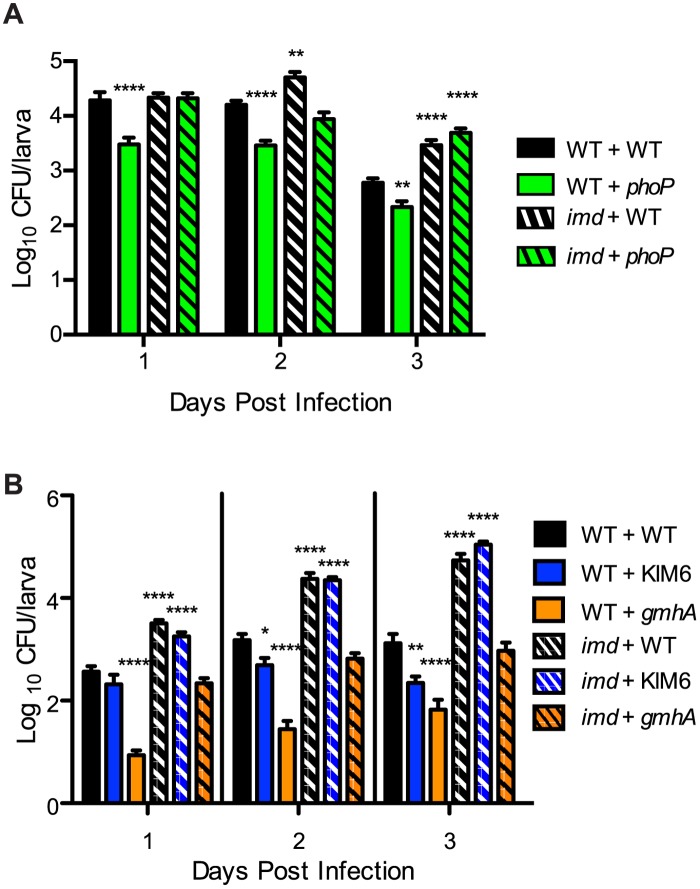
Host AMP production controls *Y*. *pestis* colonization. Wild type (WT) or *imd* mutant *Drosophila* larvae were infected with WT or mutant *Y*. *pestis* strains, and bacterial loads per larvae were monitored over three days. (A) WT (solid bars) and *imd* mutant (striped bars) fly strains were infected with WT KIM6+ (black) or the *phoP* mutant (green). Infections were performed at least in triplicate (n ≥15 for each bar) and data were pooled. (B) WT (solid bars) and *imd* mutant (striped bars) fly strains were infected with WT KIM6+ (black), KIM6 (blue), or the *gmhA* mutant (orange). Bacterial loads in WT flies were determined by plating homogenates on YSA, whereas *imd* flies were analyzed by qPCR. Infections were performed at least in triplicate (n ≥25 for each bar) and data were pooled. For both panels, means with standard error are shown. ANOVA with Dunnett's post-test was performed using KIM6+ infections of WT flies as the control, and comparisons were made for each day. ** P<0.01, **** P<0.0001.

Our data thus far indicate that resistance to AMP and ROS stress in the insect gut is important for *Y*. *pestis* to establish a stable niche. If true, then eliminating the production of stressors in the gut would rescue the colonization defects of the bacterial *phoP* and *oxyR* mutants, respectively. The beauty of using *D*. *melanogaster* as a model system is that genetic manipulation is well established and many mutants exist. AMP production in the gut is driven primarily by the IMD-Relish pathway [32,68]. We therefore obtained an *imd* mutant fly line to determine whether this mutation could restore colonization to the corresponding bacterial mutants (Fig 5). Wild type flies infected with the *phoP* mutant displayed significantly reduced levels of colonization throughout the time course (Fig 5A, solid bars), in accordance with previous data (Fig 4A and S3 Fig). In comparison, *imd* mutant flies demonstrated increased bacterial burdens (striped bars). The bacterial loads for *imd* mutants infected with wild type *Y*. *pestis* were slightly higher on days 2 and 3, as expected. Importantly, compared to infections in wild type larvae, the *Y*. *pestis phoP* mutant colonized *imd* mutant larvae to significantly higher levels throughout the time course. In fact, colonization by the *phoP* mutant was comparable to wild type *Y*. *pestis* in the *imd* mutant.

The above data indicate that host AMP production influences *Y*. *pestis* colonization. Considering our data showing that the *gmhA* mutant is defective in both biofilm production and AMP resistance, we also tested whether the severe colonization defect of the mutant could be wholly or partially rescued by the *imd* fly mutant. We also included the biofilm-deficient KIM6 strain to assess the contribution of biofilm to protection against AMPs in vivo. As shown in Fig 5B, KIM6 colonization was modestly decreased compared to wild type *Y*. *pestis* in wild type flies (solid bars), while the *gmhA* mutant defect was more severe. However, KIM6 colonization was completely rescued and was comparable to wild type *Y*. *pestis* in *imd* flies (striped bars). In contrast, colonization of the *gmhA* mutant was only partially rescued by the *imd* mutant. Bacterial loads for *gmhA* in *imd* flies were equivalent to wild type *Y*. *pestis* levels in wild type flies throughout the time course.

### Rescue of a ROS-sensitive bacterial mutant in vivo

**Fig 6:**
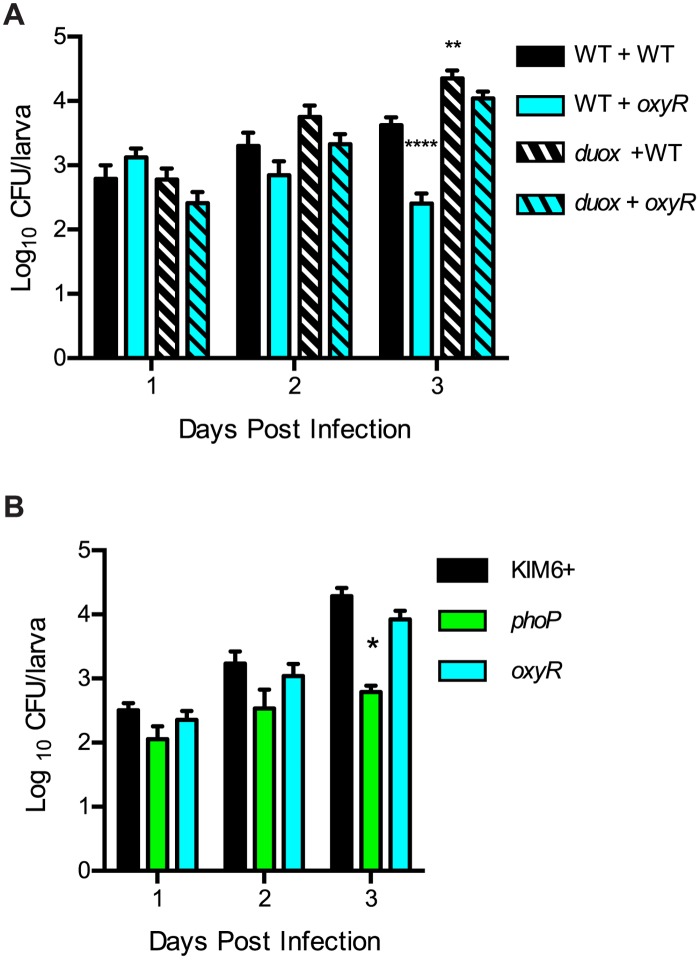
Host ROS production controls *Y*. *pestis* colonization. Wild type (WT) or *Duox* mutant *Drosophila* larvae were infected with WT or mutant *Y*. *pestis* strains, and bacterial loads per larvae were monitored over three days. (A) WT (solid bars) and *Duox* mutant (striped bars) fly strains were infected with WT KIM6+ (black) or the *oxyR* mutant (light blue). (B) *Duox* mutant flies were infected with WT KIM6+ (black), the *phoP* mutant (green), or the *oxyR* mutant (light blue). For both panels, infections were performed at least in triplicate (n ≥15 for each bar) and data were pooled. Means with standard error are shown. ANOVA with Dunnett's post-test was performed using KIM6+ infections of WT flies as the control and comparisons were made for each day. * P<0.05, ** P<0.01, **** P<0.0001.

Dual oxidase (Duox), an NADPH oxidase, is expressed by gut epithelial cells and is responsible for ROS production as a first line of defense against invading microorganisms in the gut [57]. Similar to the *imd* mutant, *Duox* mutant larvae infected with wild type *Y*. *pestis* displayed slightly higher bacterial burdens compared to wild type larvae on day 3 (Fig 6A). As expected the *oxyR* mutant yielded ~10-fold lower bacterial burden in wild type flies on day 3 compared to wild type *Y*. *pestis* (solid bars). Importantly, colonization by the *oxyR* mutant was completely restored in the *Duox* mutant, as there was no significant difference between colonization levels for the *oxyR* mutant and wild type *Y*. *pestis* strains on day 3 (striped bars). To demonstrate that the rescue of the *oxyR* mutant was specific to the mutant's ROS sensitivity, we also assessed colonization of the *Y*. *pestis phoP* mutant in the *Duox* mutant (Fig 6B). Though wild type *Y*. *pestis* and the *oxyR* mutant grew equally well, the *phoP* mutant retained its modest colonization defect in *Duox* flies.

### Colonization of fleas

Given that *Y*. *pestis* strains that are sensitive to AMP and ROS defenses are attenuated in the *Drosophila* infection model, we expected to see similar defects for early colonization events in fleas. To test this hypothesis, we infected *O*. *montana* and *X*. *cheopis* fleas with wild type *Y*. *pestis* or representative mutant strains and monitored colonization over 7 days. The *oxyR* mutant was chosen for its ROS sensitivity, while the *gmhA* mutant was chosen as a representative of the AMP-sensitive class of mutants. Fleas were fed an infectious blood meal containing 10^9^ CFU, after which bacterial loads were assessed on days 1 and 7.

**Fig 7:**
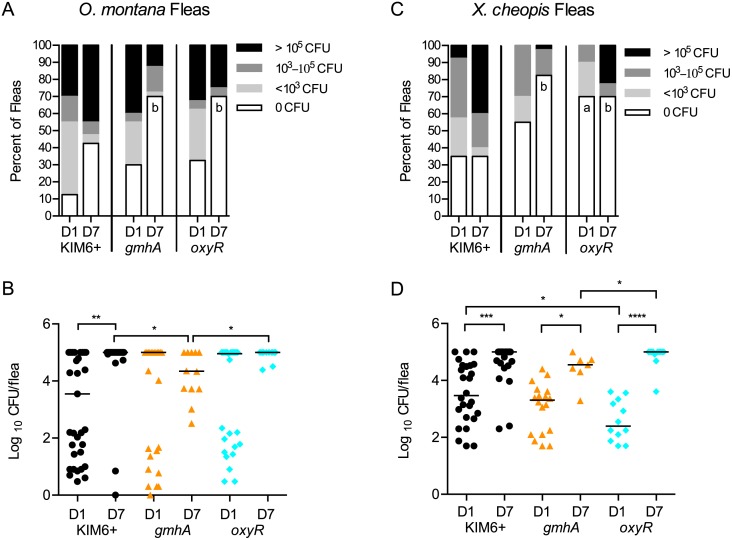
Colonization of *Y*. *pestis* mutants in *O*. *montana* and *X*. *cheopis* fleas. *O*. *montana* (panels A-B) or *X*. *cheopis* (panels C-D) fleas fed on blood containing 1x10^9^ CFU of the indicated *Y*. *pestis* strains. On days 0, 1 (D1) and 7 (D7) post-infection, fleas were mechanically disrupted and enumerated on heart infusion agar. Day 0 data are shown in S2 Fig. Panels A and C: Fleas were grouped according to their bacterial burden and are represented as a percentage of the total population for that bacterial strain and day. Minimum limit of detection was 1 CFU; Maximum limit of detection was 10^5^ CFU. Data shown were collected in 1 (A-B) or 2 (C-D) independent trials; n = 40 fleas per strain per day. Data were analyzed by Chi-square test with Bonferonni post-test, comparing each mutant to KIM6+, with a significance level of P<0.025. "a" denotes significant comparisons on day 1; "b" denotes significant comparisons on day 7. Panels B and D: Analysis of only infected fleas from Panels A and D; fleas with 0 CFU not included. Bacterial load from each infected flea is displayed, and the bar in each lane indicates the population median. A Kruskal-Wallis non-parametric analysis with Dunn's post-test for multiple comparisons was performed, and significant pairwise comparisons are indicated: * P<0.05, ** P<0.01, *** P<0.001, **** P<0.0001.

For *O*. *montana* fleas ingesting the wild type strain, KIM6+, we observed an infection rate of ~90%, with nearly half of the infected fleas harboring fewer than 10^3^ CFU (Fig 7A). The infection rates of *gmhA* and *oxyR* mutants were not detectably different from KIM6+ on day 1. Additionally, the titers of infected fleas were similar, indicating that both mutants initially colonized fleas as well as wild type (Fig 7B and S2 Fig). By day 7, the KIM6+ infection resolved into the characteristic bimodal distribution [53] wherein a significant portion of the population has cleared the infection (~40% of fleas), and the majority of infected fleas had >10^5^ CFU (Fig 7A). Both mutants displayed a bimodal distribution as well; however, the percentage of fleas (>60%) that had cleared the mutant infections was significantly higher than for wild type. For those fleas that remained colonized, growth of the mutants was apparent, and in fact, titers of the *oxyR* mutant were similar to wild type (Fig 7B). In contrast, growth of the *gmhA* mutant was significantly reduced.

Similar to *O*. *montana*, the wild type infection of *X*. *cheopis* fleas resolved into the characteristic bimodal distribution pattern by day 7 (Fig 7C). Again, both *oxyR* and *gmhA* mutants were cleared from fleas by day 7 at a significantly higher rate compared to the wild type strain, and the trends in bacterial growth were similar to the results observed during infection of *O*. *montana* (Fig 7D).

## Discussion

The majority of research on the transmission of *Y*. *pestis* from the flea vector has focused on the classical biofilm-dependent model using the Oriental rat flea *X*. *cheopis*. The development of a transmissible infection in this model is facilitated by blockage of the flea proventriculus by a *Y*. *pestis* biofilm. This process may take 1–2 weeks, and blocked fleas starve to death soon after forming a transmissible biofilm [53]. It has been suggested that the prolonged incubation period necessary for blockage of the flea (extrinsic incubation) is not sufficient to explain the speed at which *Y*. *pestis* outbreaks occur [69]. In addition, many flea species can efficiently transmit an infection even though biofilm blockage is not prevalent [1,5,8,10,11], and some of these fleas, including *O*. *montana*, are important vectors of plague in North America. Additionally, plague transmission appears to be of a cyclic nature, whereby epizootic episodes, in which massive die-offs occur in susceptible host populations, are interspersed with quiescent periods (inter-epizootic), in which there is little to no detectable vertebrate host death. At present, there is no simple explanation to account for the disease cycles; however it is possible that both long-term persistence and rapid spread could arise from a combination of the classical biofilm-dependent transmission mechanism and EPT, and could depend on a number of factors including susceptibility of both the vertebrate host and the flea vector to bacterial colonization. However, while much work has gone into defining interactions between *Y*. *pestis* and its vertebrate hosts, relatively little is known about the interactions between this bacterium and its vectors.

The discovery of EPT, in which the flea is able to transmit *Y*. *pestis* immediately after becoming infected and without the need for biofilm, raises a number of questions about the relationship between *Y*. *pestis* and its insect vector. Microarray data for bacteria harvested from blocked fleas indicated that the transcriptional regulator *phoP* was induced, and further work demonstrated that although wild-type levels of bacteria were recovered from fleas four weeks post-infection with a *phoP* mutant, fewer fleas became blocked [26,27]. Furthermore, transcriptome analysis of infected fleas demonstrated that genes required for ROS production and detoxification were induced, and moreover that fleas infected with *Y*. *pestis* lacking the transcriptional regulator *oxyR* had lower bacterial burdens [25]. These data imply that the interplay between the gut innate immunity and the counteractive *Y*. *pestis* resistance mechanisms likely influence colonization events in fleas.

*D*. *melanogaster* provides a premier genetic system, which has been used to model a variety of host-pathogen interactions. The *Drosophila* genome is well characterized, and a wealth of genetic tools and mutant strains are available to study microbe-host interactions. In this study, we present evidence that *D*. *melanogaster* is a suitable model for colonization of the flea by *Y*. *pestis*. Using a non-invasive infection model [31], we were able to orally infect *D*. *melanogaster* larvae with *Y*. *pestis*. Ingested bacteria are maintained as a stable population within the neutral segment of the larval anterior midgut (Fig 2). Importantly, the infection does not become invasive or detrimental to larval development and survival. These observations are similar to those of infected adult fleas, whereby bacteria appear to be non-invasive and confined to the flea midgut [70]. Colonization of flea larva has not been rigorously examined, so correlates between the larval and adult stages of fleas cannot be made. Notably, production of AMPs and ROS is an innate immune response conserved across insects and vertebrate animals, though details such as the type of AMP or the magnitude of its expression may change; therefore *D*. *melanogaster* has emerged as a leading model for study of innate immunity and gut homeostasis [32,56,67,71–76].

Biofilms play an important role in the blockage-dependent model of plague transmission; however flea colonization can occur in the absence of biofilms, and EPT is biofilm independent. Towards validating the usefulness of the *Drosophila* model system in early-phase colonization studies, we assessed the ability of known biofilm-deficient strains to colonize fly larvae. Both the *pgm* mutant KIM6 and the *hmsH* mutant, which are completely biofilm deficient, were able to colonize the fly larvae albeit at slightly reduced levels compared to wild type. This suggests that the *Drosophila* model may be able to detect interactions, which do not necessarily rely on biofilm formation but are required for colonization, and this model may therefore be helpful in elucidating biofilm-independent flea colonization processes. Furthermore, we found that the small difference in colonization of fly larvae by the biofilm-deficient KIM6 strain could be partially complemented when the bacteria were grown in the *imd* mutant fly. This suggests that while biofilms are not essential for fly colonization, they may aid in persistence of the bacteria in a manner similar to that of fleas, where it is thought that the large bacterial clumps are more likely to resist the peristaltic forces of the gut ([61], and/or bacteria within these clumps may experience some protection from the immune response.

Interestingly, the *gmhA* mutant that has been shown to be deficient in biofilm formation and in colonization of the *C*. *elegans* model system [62], showed a much more severe colonization defect than any of the other mutant strains we tested in the *Drosophila* and flea models (Fig 4A). In addition, when the *gmhA* mutant was exposed to the cationic AMP polymyxin B (S5 Fig), growth was drastically reduced, indicating that the severe colonization defect may be due to a combination of factors. The fact that the *imd* fly mutant could not completely rescue the *gmhA* mutant's colonization (Fig 5B) indicates that susceptibility to AMPs is not the only culprit for this strain's defect. It appears that the role of GmhA in vivo is complex and that the state of the LOS impacts more than biofilm production and AMP resistance. Notably, the *gmhA* mutant does not have a gross growth defect, as it grows normally in vitro in HIB medium (data not shown and [62]). Furthermore, the mutant was capable of surviving exposure to hydrogen peroxide in an in vitro test for sensitivity to oxidative stress (S6 Fig), suggesting that while the mutant phenotype is complex, it is not uniformly sensitive to all stressors. Further work will be necessary to unravel the additional roles that LOS biosynthesis plays in resisting host immunity. Importantly, the *Drosophila* model was able to distinguish between biofilm-deficient mutants and a mutant with a combination of colonization defects.

The *oxyR* mutant, lacking expression of peroxidases and catalases that are essential in defending against ROS, was unable to efficiently colonize fly larvae. This colonization defect extended to fleas, wherein the *oxyR* mutant was cleared at a higher rate by both *O*. *montana* (Fig 7A) and *X*. *cheopis* fleas (Fig 7B)[25]. We were able to rescue the colonization defect of the *oxyR* mutant by using a *Duox* mutant fly line, which is defective for ROS production in the gut (Fig 6). These data agree with previous work showing that ROS production genes were induced upon infection, that ROS levels were higher in infected fleas, and that addition of antioxidants to the blood meal enhanced *Y*. *pestis* growth in fleas [25].

Previous work using *X*. *cheopis* fleas demonstrated that *phoP* is induced in blocked fleas [26], suggesting that some part of its regulon may be important for colonization. Subsequent work demonstrated that although the *phoP* mutant was able to colonize *X*. *cheopis* fleas as well as wild type, it was severely compromised in blocking those fleas and showed increased sensitivity to polymyxin B and the insect AMP cecropin A [27]. The role of the PhoP/Q system remains unclear, however, since there are differences in the PhoP regulon depending on the environmental conditions [27,29]. Recent work indicates that PhoP contributes to tolerance of mildly acidic conditions that might be encountered in the flea gut [29], but investigations toward PhoP’s contribution to AMP resistance in fleas had yielded conflicting data [27,28]. In one study, deletion of *ugd* and *pbgP*, which are involved in aminoarabinose synthesis, still resulted in fleas that were colonized and blocked, similar to wild type *Y*. *pestis*, after 28 days [27]. In later work, it was found that mutations in *arnB* (*pbgP*) as well as *galU*, which is also required for the aminoarabinose modification of lipid A, led to reduced colonization of fleas after 4 days [28]. The methods used differ between these two studies, so further work is necessary to resolve the role of lipid A modification during flea infection and to confirm that it is a PhoP-mediated phenomenon. It is also possible that other uncharacterized putative PhoP targets [29] mediate AMP resistance through as yet unknown mechanisms of LOS modification. Alternatively, AMP protection may be afforded through PhoP-mediated control of biofilm production and/or modification, a notion that is supported by the fact that a *phoP* mutant makes less-cohesive biofilms compared to wild type bacteria *in vivo* [27]. Furthermore, protection from AMPs by biofilms has been demonstrated for several pathogens, including *Staphyloccoci*, which produce a poly-N-acetylglucosamine-dependent biofilm similar to *Y*. *pestis* [77–83].

The link between PhoP and biofilm production in *Y*. *pestis* is not yet clear; however there is data from *gmhA*, *yrbH*, and *waaA* mutants, which produce defective LOS, establishing a link between defects in the cell surface and defective biofilms (Fig 5 and [62,84]). Our data add to those observations by demonstrating that a *phoP* mutant yields reduced bacterial burdens in the fly model. Furthermore, we demonstrated that colonization by the *phoP* mutant was rescued in the *imd* mutant fly (Fig 5A). In *Drosophila*, the IMD pathway controls production of several classes of AMPs in the gut [56,68,85]. These data indicate that AMP production plays a role in controlling *Y*. *pestis* infection. It is possible that the mechanisms of AMP resistance differ during flea infections compared to fly infection, and it remains to be determined whether changes such as the aminoarabinose modification of lipid A contribute to AMP resistance in flies. Nevertheless, the rescue of the colonization defects of both the *phoP* mutant and the KIM6 biofilm mutant by the *imd* fly reinforces the link between PhoP and biofilms and implies that control of biofilm production by PhoP might be one mechanism by which *Y*. *pestis* resists AMP stress in the insect gut.

Though the mechanism by which PhoP facilitates AMP resistance in fleas is unclear, it is noteworthy that both *phoP* and *gmhA* are linked to biofilm production and AMP resistance, and mutation of either gene abrogates blockage of the flea proventriculus while still permitting chronic infection of the midgut [27,62]. Interestingly, AMP production in the proventriculus (or cardia) of several fly species is higher than in the midgut [56,86,87]. If true in fleas, then the proventriculus might present a more hostile, antimicrobial environment than the midgut. At present, this issue remains unresolved due to a lack of information regarding the identity of specific AMPs and the timing, magnitude, and locale of their production within infected fleas.

Overall, our data support the notion that resistance to AMP and ROS plays an important role in vector colonization. While we do not know which components of the OxyR and PhoP regulons are responsible for efficient colonization, our new *Drosophila* model system will facilitate future studies aimed at identifying the specific components of the OxyR and PhoP regulons, as well as other genes that participate in insect colonization and ultimately transmission of flea-borne diseases.

## Supporting Information

S1 Appendix Supplementary Methods. (DOCX)

S1 Fig Quantitative PCR (qPCR) validation. Wild type *Drosophila* larvae infected with WT *Y*. *pestis* were homogenized in PBS and a portion of that suspension was plated to determine CFU counts. The remainder of the suspension was subjected to DNA extraction followed by qPCR analysis. Actual CFU counts (from plating) were plotted against estimated CFU counts generated from qPCR data. A linear regression was performed to fit the data points from 15 individual larvae. **(A)** qPCR validation using *Taq*Man method. **(B)** qPCR validation using SYBR Green method.
(EPS)

S2 Fig Infecting doses of *Y*. *pestis* strains in *O*. *montana*. *O*. *montana* fleas fed on blood containing 1x10^9^ CFU of the indicated *Y*. *pestis* strains. Uniform infecting doses between strains was verified by enumerating the bacterial load in 5 fleas just after feeding on infected blood. Fleas were mechanically disrupted and enumerated on heart infusion agar. Bacterial load from each infected flea is displayed, and the bar in each lane indicates the population mean. ANOVA with Tukey post-test for multiple comparisons was performed, and no significant differences were found.
(EPS)

S3 Fig Complementation of *Y*. *pestis* mutants. Fruit fly larvae were infected WT KIM6+ *Y*. *pestis*, the indicated mutants, or the complemented mutants, and bacterial survival was evaluated. Means and standard error are shown for each set of infections. Infections were performed at least in triplicate. Total larvae per strain per day: n = 45 for the *oxyR* and *gmhA* sets; n = 30 for the *phoP* set. ANOVA with Dunnett post-test was done using KIM6+ infections as the control. ** P<0.01, **** P<0.0001.
(AI) Click here for additional data file.

S4 Fig Biofilm formation of the *Y*. *pestis* mutants. *Y*. *pestis* cultures were grown overnight on borosilicate with shaking before being stained with crystal violet. The dye was solubilized using acetic acid and biofilm formation was quantified at A_550_. Each strain was cultured independently in quadruplicate. The data were averaged and standard error is shown. ANOVA with Dunnett post-test was done to compare mutants to the KIM6+ control. ** P<0.01, *** P<0.001, **** P<0.0001.
(EPS)

S5 Fig Polymixin B sensitivity of *Y*. *pestis* mutants. *Y*. *pestis* cultures at a starting density of ~ 10^8^ CFU/mL were serially diluted and plated on HIA containing the indicated concentration of polymyxin B. The plates were incubated at room temperature for two days, and colonies were counted to determine bacterial sensitivity.
(EPS)

S6 Fig Hydrogen peroxide sensitivity of *Y*. *pestis* mutants. *Y*. *pestis* cultures were mixed with hydrogen peroxide to give a final concentration of 10^7^ CFU/mL in 2.5 mM H_2_O_2_. After 20 minutes of incubation at 26°C, bacteria were diluted into HIB to quench the reaction and then enumerated by plating on HIA. Reactions were performed in triplicate and means with standard error are shown. ANOVA with Dunnett post-test was done to compare all strains to the KIM6+ control. * P<0.05.
(EPS)
