# Supplementary material for: Resistance to Innate Immunity Contributes to Colonization of the Insect Gut by Yersinia pestis
Source: PLoS One. 2015 Jul 15;10(7):e0133318. doi: 10.1371/journal.pone.0133318 (PMC4503695; doi:10.1371/journal.pone.0133318)
Supplement: S1 Appendix — (DOCX) [file pone.0133318.s001.docx]

**Supplementary Methods:**

**qPCR validation.** To validate the qPCR methods and verify correlation to CFU counts, larvae were first homogenized in PBS and a portion of the suspension was plated on YSA to determine CFU counts. Then the remainder of the suspension was used to extract the DNA, followed by qPCR analysis as described in Materials and Methods of the main text. The correlation curves for CFU counts vs *Taq*Man qPCR and CFU counts vs SYBR Green qPCR are shown in S1 Fig.

**Polymyxin B sensitivity assay.** *Y. pestis* were grown overnight in HIB and adjusted to a starting concentration of 10^8^ CFU/ml. Cultures were serially diluted in PBS and plated onto HIA containing the indicated concentration of polymyxin B before incubation at room temperature for 2 days. Colonies were counted to determine the bacterial survival rate. Data shown are pooled from independent experiments: 3 trials for KIM6+, 2 trials each for *phoP* and *gmhA*, 1 trial each for *oxyR* and all three complemented mutants.

**ROS sensitivity assay.** Overnight cultures were collected in exponential phase and bacterial density calculated using OD_600_ values. Each culture was diluted in PBS to reach a concentration of 2x10^7^ CFU/ml. An aliquot of each culture was combined with an equal volume of 5 mM hydrogen peroxide (freshly diluted into PBS) to reach a final bacterial concentration of 10^7^ CFU/ml in 2.5 mM hydrogen peroxide. Preparations of bacteria in PBS only were used for controls. All samples were incubated at 26°C for 20 minutes with moderate agitation in a roller drum. Each sample was serially diluted in HIB and plated onto HIA for enumeration of bacterial survival.

**Biofilm assay.** *Y. pestis* strains were inoculated into 1 ml of HIB in borosilicate tubes and incubated at room temperature with shaking overnight. Medium was then removed and cells were stained with 0.1% crystal violet. The dye was rinsed gently three times with water and then solubilized with 33% acetic acid. Crystal violet staining was measured at A_550_. Assays were done in quadruplicate for each strain. Statistical analysis was performed using one-way ANOVA with Dunnett's test.
